# Evaluation of urology trainee preferences in didactic education: a choice-based conjoint analysis

**DOI:** 10.3389/fmed.2023.1144092

**Published:** 2023-07-06

**Authors:** Yi Li, Kyle Spradling, Isabel Elaine Allen, Simon Conti, Lindsay A. Hampson

**Affiliations:** ^1^Department of Urology, University of California, San Francisco, San Francisco, CA, United States; ^2^Department of Urology, Stanford University, Palo Alto, CA, United States; ^3^Department of Epidemiology and Biostatistics, University of California, San Francisco, San Francisco, CA, United States

**Keywords:** didactics, trainee preferences, resident education, collaborative learning, conjoint analysis

## Abstract

**Purpose:**

Didactic lectures are a commonly used educational tool during urology residency training. Recently, there has been a rapid introduction of online, collaborative didactics as a new model for resident teaching. The aim of this study is to determine which attributes of didactics education are most preferred by contemporary urology trainees.

**Methods:**

Urology trainees were invited to complete an online choice-based exercise assessing combinations of four attributes associated with didactics education: mode of communication, learning style, presenter credentials, and curriculum design. The survey was distributed via social media platforms and the Urology Collaborative Online Video Didactics (COViD) website. A choice-based conjoint analysis was used to identify how the trainees valued different combinations of didactic education.

**Results:**

Seventy-three trainees completed the conjoint analysis exercise. Mode of communication was rated as significantly more important than curriculum design (relative importance 28.6% vs. 19.9%). Overall, the majority preferred online/virtual presentations to in-person presentations. Respondents preferred national experts to faculty members from their local institutions, and preferred cased based lectures to didactics style lectures. A nationally standardized curriculum was also preferred over curriculum designed by local institutions. Finally, when segmented by level of training, there was increased preference for overall favored options as PGY year increased.

**Conclusion:**

This conjoint analysis shows clear preference by trainees for online, recorded didactics, nationally standardized with national experts, and preferably in a case-based format. Academic societies in urology and program directors should consider utilizing the shared experience of previously created collaborative online lectures in developing future didactic curriculum that can meet the needs of current trainees.

## Introduction

The traditional model for didactic education in urological residency training programs has relied on lectures by local faculty and self-directed textbook learning. Each training program creates its own curriculum, relying on the faculty expertise within their program to provide the necessary educational expertise. This education system inherently contains a high degree of heterogeneity and results in silos of knowledge limited to each program (1).

The COVID-19 pandemic brought about drastic changes in education as social distancing regulations encouraged changes to online classes at all levels of learning (2). Urology residency programs similarly pivoted to online teaching. The Collaborative Online Video Didactics (COViD) Lecture Series was launched in March of 2020 and featured volunteer experts from around the world giving twice daily online lectures to students, residents, fellows, and other practicing urologists (3). The program was received highly positive feedback and has led the way to other collaborative online teaching series at both a resident and fellowship level (4, 5).

Since the development of these programs, there have been calls for professional urologic societies to standardize resident didactic teaching utilizing this new collaborative online model (6). While the collaborative online lectures were well received, the question still remains whether learners truly prefer a standardized online lecture format with recorded option for future viewing by national experts from different institutions, or in-person lectures by faculty at their local institution, designed and implemented independently by each institution. This study aims to utilize a conjoint analysis approach to query urology trainees to determine their preferences regarding urology didactics. We hypothesize that urology trainees will prefer standardized online, case-based lecture by national experts.

## Participants and methods

After institutional review board approval, urology trainees of all levels were invited to complete an online choice-based conjoint analysis exercise via social media platforms (Twitter) and the Urology Collaborative Online Video Didactics (COViD) website. A total of 636 surveys were initiated via the Sawtooth website (indicating that someone clicked on the link to take them to the survey) between June 2020 and June 2022. The conjoint analysis was designed to assess combinations of four attributes associated with didactic education, including: (1) mode of communication (online/recorded vs. in-person) (2) learning style (case-based vs. lecture-based) (3) presenter credentials (national expert vs. faculty at local institution); and (4) curriculum design (standardized and shared vs. designed and implemented independently by each institution). These attributes were determined by expert consensus by the author group as the differences that would be most important in delineating the dichotomy between online and in person learning.

Respondents completed a brief questionnaire on demographic information including gender, age, PGY level (Medical student, PGY 1–3, PGY 4–6, fellow), daily commute time (<30 min, 30–60 min, >60 min), and end of workday time (before 5 PM, 5–7 PM, 7–9 PM). The conjoint analysis was developed and administered using the Sawtooth Discover Web Application (Sawtooth Software, Inc., Provo, UT).

**Figure 1:**
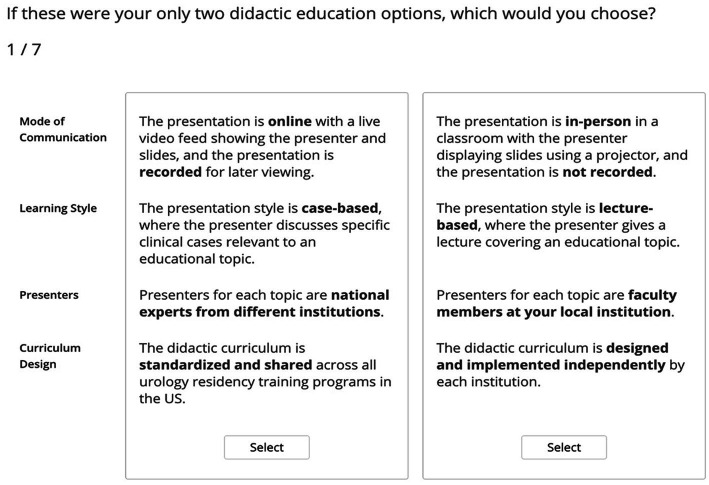
Example of conjoint analysis scenario survey question which incorporates one of each of the attributes being tested. Selection of multiple scenarios allows for assessment of both preference and weight (importance) of each attribute as the attributes are varied in the questions.

Participants were given eight questions regarding attribute levels and asked to rank the desirability of each attribute (Undesirable, Somewhat Desirable, Very Desirable). They then answered seven choice-based conjoint exercise questions in which they were offered two hypothetical treatment scenarios and prompted to choose their preferred option. The scenario questions included random combinations of didactic education attributes and were designed to assess how much importance trainees placed on each attribute (Figure 1).

The conjoint analysis was performed using Market Simulator software within the Sawtooth Discover Web Application to predict the proportion of participants who would select each lecture option. The details behind this approach have been previously published (7). Conjoint analysis utilities of each attribute level were calculated. A conjoint analysis utility is a numerical score assigned to each attribute and used in the analysis coding to measure how much an attribute influences a participant’s decision. By measuring the range in utility of each attribute, the relative importance of each treatment attribute was calculated. Demographic characteristics were selected for additional stratified analyses, and confidence intervals (CIs) between subgroups were calculated. Statistical significance was determined based on the degree of overlap of the 95% CIs between subgroups. Comparisons were done using MedCalc online to calculate *p* values (8).

## Results

Of the 636 survey clicks, 75 respondents started the exercise with 73 completing all scenarios. Sixty three percent of respondents were male, and 79% were resident level trainees. Twenty percent of respondents were international, and 84% were either in fellowship or plan to pursue fellowship after residency training. Eighty two percent of trainees preferred more than an hour per week of didactic lectures. Nearly all respondents (97%) were ≤ 40 years of age. Full demographic characteristics are listed in Table 1 including self-reported daily commute, average hour leaving the hospital, desired hours of didactic lectures per week.

**Table 1:** Demographics of survey respondents.

Characteristic (*N* = 75)	*N* (%)
Age	
<25	0 (0)
25–30	33 (44)
31–35	30 (40)
36–40	10 (13)
>40	2 (3)
Gender	
Male	47 (63)
Female	29 (37)
Other	0 (0)
Level of training	
Junior resident (PGY 1–3)	30 (40)
Senior resident (PGY 4–6)	29 (39)
Fellow	16 (21)
AUA section	
Northeast	3 (4)
New England	2 (3)
New York	1 (1)
Mid-Atlantic	8 (11)
Southeast	1 (1)
South Central	3 (4)
North Central	2 (3)
West	40 (53)
International	15 (20)
Plans after residency	
Fellowship	62 (84)
Practice	12 (16)
Daily commute	
<30 min	40 (53)
30–60 min	22 (29)
>60 min	13 (17)
Average hour Leaving Hospital	
Before 5 PM	7 (9)
5–7 PM	52 (69)
7–9 PM	16 (21)
After 9 PM	0 (0)
Desired hours per week of didactics	
<1	1 (1)
1	13 (17)
2	39 (40)
3	16 (21)
4	7 (9)
>4	8 (11)

The conjoint analysis rated respondent attribute importance. Mode of communication (relative importance 28.6, 95% CI 23.9–33.3) was rated as significantly more important than curriculum design (19.9, 95% CI 16.2–23.6) (*p* = 0.04). Learning style (26.2, 95% CI 21.6–30.9) (*p* = 0.06) and presenter (25.3, 95% CI 20.9–29.7) (*p* = 0.57) ranked in between, without any significant difference.

**Figure 2:**
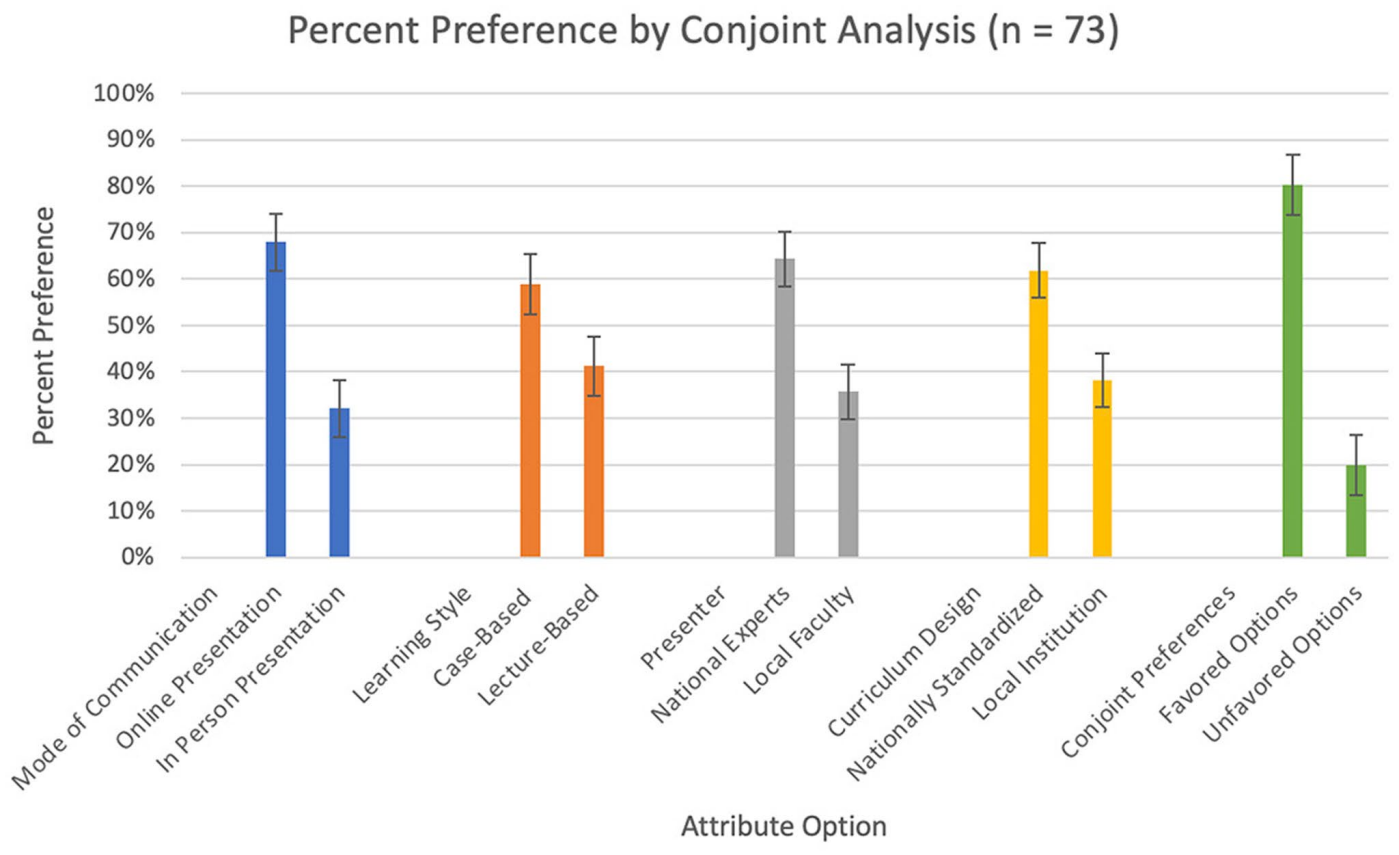
Conjoint analysis results, percent preference based on mode of communication, learning style, presenter, and curriculum design. The last category, conjoint preferences shows the percent preference if all of the favored options are compared to all of the unfavored options. Error bars are 95% CI calculations based on the conjoint analysis.

**Figure 3:**
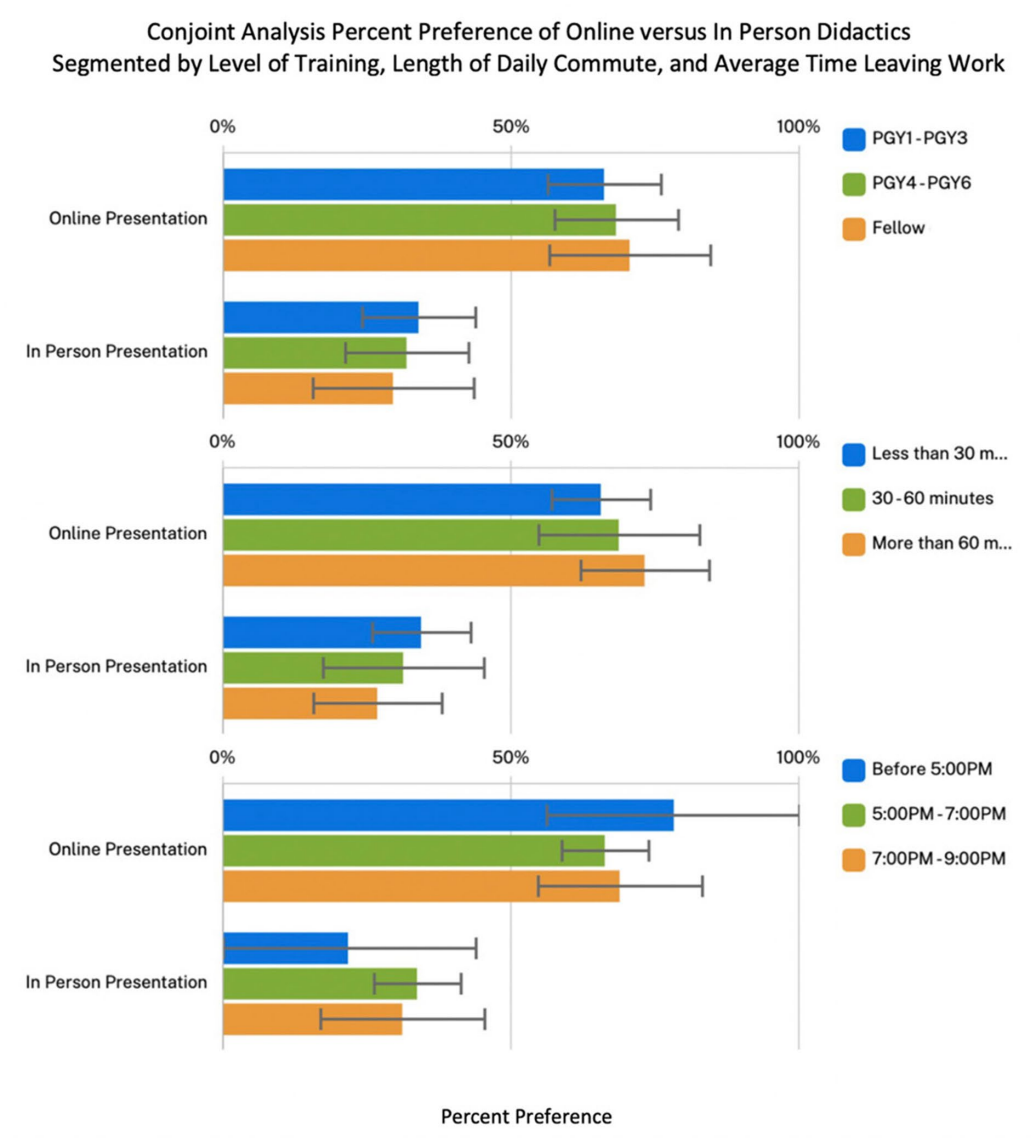
Conjoint analysis of percent preference between online lectures versus in person lectures as segmented by level of training, length of commute from work, and average hour leaving work. Error bars are 95% CI based on the conjoint analysis.

Conjoint analysis showed that respondents had a strong significant preference for online and recorded didactics (67.9, 95% CI 61.7–74.0) compared to in-person lectures (32.1, 95% CI 26.0–38.3) (*p* < 0.01) (Figure 2). There was no significant difference noted in preference when segmented by level of training, hour leaving hospital, length of commute, or any other demographic variables (Figure 3).

Respondents also significantly preferred case-based didactics (58.9, 95% CI 52.6–65.3) to lecture-based didactics (41.2, 95% CI 34.7–47.6) (*p* = 0.03). There was also a significant preference for national experts (64.3, 95% CI 58.3–70.3) over local faculty (35.7, 95% CI 29.7–41.8) (*p* < 0.01). There was a significant preference for a nationally standardized curriculum (61.8, 95% CI 56.0–67.7) to locally designed and implemented curriculum (38.2, 95% CI 32.3–44.1) (*p* < 0.01) (Figure 2).

When selecting between all four favored attributes compared to the four unfavored options, the favored options (80.2, 95% CI 73.7–86.8) was significantly preferred over the unfavored options (19.8, 95% CI 13.2–26.3) (*p* < 0.01). When segmented by level of training, junior residents had a higher preference level for the unfavored options (24.1, 95% CI 13.4–34.8) when compared to fellows (7.28, 95% CI 1.90–12.7) (*p* < 0.01).

## Discussion

The transition from the classic, in-person lecture style of resident didactic education to an online, standardized didactic curriculum was switched due to the COVID-19 pandemic, giving trainees exposure to an alternative method of receiving didactic education (4, 9–11). In this study, trainees were queried via online conjoint analysis as to their preferences regarding didactic lectures. All levels of trainees showed clear preferences within all four attributes of didactic lectures evaluated.

Given that this study was conducted following the implementation of online recorded lecture series such as the Urology Collaborative Online Video Didactic Series and the EMPIRE lecture series, respondents had had exposure to an online lecture experience, and could compare directly with their prior experiences of local in-person lectures (3, 4). Overall, respondents strongly preferred online, recorded lectures to in-person, non-recorded lectures. In addition, participants valued the communication mode (online vs. in-person) attribute the most, indicating a clear desire for online and recorded lectures. Within the survey we inquired about length of commute and average hour leaving work, with the idea that trainees who must travel farther, or work later, may have a stronger preference for a recorded lecture that can be reviewed at a more convenient time. However, there was not a significant difference found when segmented by these categories, nor was there any difference noted when segmented by level of training, showing that this preference really did not vary significantly across individuals and was widely held by the vast majority.

Currently, the collaborative online teaching series have stopped as clinical and surgical volume have returned to pre-pandemic levels. However, given the ongoing COVID risk and convenience of virtual conferences, many urology programs have continued online conferences, didactics, and journal clubs. The Society of Academic Urology (SAU) published best practice guidelines recommending the continuation of “didactics and conferences via video and teleconferencing media” (12). Our survey findings show that trainees also prefer this modality of lectures (Figure 3).

In our study, respondents also had a clear preference for case-based presentations as compared to lecture-based presentations. Case-based learning increases learner engagement by creating intrinsic goals for the learner (solving a riddle) and applying context to the material. This style of lecture helps to optimize the “germane load” as described in cognitive load theory (13). This directly counters one of the most common criticism of online lectures, namely the lack of learner engagement and increased distractions compared to in person teaching. Case-based teaching has been shown to be preferred by trainees when implemented at urology residency programs (14). Analysis of post-lecture evaluations in the UrologyCOViD series also showed higher ratings for case-based lectures compared to traditional guidelines based, surgical technique based, and practice update style lectures (15). Respondent preference here is noted to be consistent with these prior studies and shows why case-based teaching models are so important with regards to urology didactic teaching.

Respondents also showed a significant preference for national expert presenters compared to local faculty. When segmented by level of training, there was a trend for fellows to have a stronger preference for national experts compared to residents, but this was not statistically significant. This trend may be explained by a need for a higher level of expertise in teaching when the trainee reaches a higher level. Additionally, junior residents may feel more comfortable with local faculty as they may be less intimidated and be able to ask more questions. Local faculty lectures also may be able to provide more context for junior learners, rather than relying on assumptions of level of understanding and exposure to a topic. Access to national experts was noted to be one of the desirable aspects of collaborative lecture programs (3).

Finally, respondents showed a preference for a nationally standardized curriculum over a curriculum designed and implemented by their local programs. This finding supports calls for professional societies such as the American Urological Association (AUA) or the Society for Academic Urology (SAU) to create a standardized didactic education tools for resident learning (6). Currently, the AUA offers a junior resident level in-person course, as well as the online Core Curriculum. These programs are limited by accessibility and limited breadth, which may be improved by incorporating the lecture libraries of programs such as UrologyCOViD and EMPIRE.

When segmented by level of learning, there was a significant difference in preference between fellows and junior residents, with fellows having a stronger preference for the favored options compared to junior residents. It is possible that this stronger preference may be related to a desire for higher levels of teaching with national expertise. We can also infer that a fellow’s prior learning experiences may have a more significant impact on their preference here.

This study has some significant limitations, specifically related to the anonymous survey nature of the study. The survey was distributed via social media and as a link from the UrologyCOViD lecture website. This distribution method creates a non-response bias as the respondents will be trainees who are already utilizing online learning platforms such as UrologyCOViD and “MedTwitter.” It is possible that the preferences reported are of those who are already facile with online learning.

The Sawtooth website also limits the ability to get an accurate assessment of response rate for the survey, as each time the link is clicked, a new survey is created. Therefore, if one individual clicked on the link multiple times before finally completing the survey, this creates an artificially high “incomplete” survey number. In comparison to the 2020–2021 AUA census, where 324 residents and 145 fellows responded, our survey response rate was approximately 18 and 11% of the national AUA census rate, respectively. We feel that this is a reasonable sampling of trainees.

One strong point of this study, however, is that nearly all of the individuals who actually started the survey completed it. The strengths of this study includes its conjoint analysis nature which allows for assessment of attribute importance in addition to preference.

Utilization of trainee-based input on didactic education has been well established, as surveys and evaluations are often utilized to improve on education methods (16). This study solicits input from trainees regarding their education needs. With generational changes to learning styles, as well as new advances in technology, it is important to continue to query learners regarding their preferred learning modalities, and to try to implement them where possible to optimize the education quality of our trainees. Beyond analysis of learner reaction and preference to this learning style, the next steps are to show that learner knowledge retention and ultimately, readiness for clinical practice is improved with online didactics. Future studies are underway to evaluate learner engagement and knowledge retention outcomes.

## Conclusion

The rapid familiarization of urologic trainees with teleconferencing media and online didactics as a result of the COVID-19 pandemic has led to re-evaluation of the optimal learning formats for residents and fellows. The results of this conjoint analysis show clear preference by trainees for online, recorded didactics, nationally standardized with national experts, and preferably in a case-based format. Academic societies in urology and program directors should consider utilizing the shared experience of previously created collaborative online lectures in developing future didactic curriculum that can meet the needs of current trainees.

## Data availability statement

The raw data supporting the conclusions of this article will be made available by the authors, without undue reservation.

## Ethics statement

Ethical review and approval was not required for the study on human participants in accordance with the local legislation and institutional requirements. Written informed consent for participation was not required for this study in accordance with the national legislation and the institutional requirements.

## Author contributions

KS and SC were involved in creation of the conjoint analysis and implementing data collection. YL, IA, and LH participated in data analysis and manuscript preparation. All authors involved in editing and approval of final manuscript.

## Funding

This project was supported by an educational grant sponsored by Sawtooth Software, Inc. who provided free access to software in order to perform the conjoint analysis.

## Conflict of interest

The authors declare that the research was conducted in the absence of any commercial or financial relationships that could be construed as a potential conflict of interest.

## Publisher’s note

All claims expressed in this article are solely those of the authors and do not necessarily represent those of their affiliated organizations, or those of the publisher, the editors and the reviewers. Any product that may be evaluated in this article, or claim that may be made by its manufacturer, is not guaranteed or endorsed by the publisher.
